# Citizenship-Oriented Care: Increasing Voting Participation in a Mental Health Trust

**DOI:** 10.1192/bjo.2025.10255

**Published:** 2025-06-20

**Authors:** Cissy Atwine, Emily Cobb, Oliver Dale

**Affiliations:** Sussex Partnership NHS Foundation Trust, Sussex, United Kingdom

## Abstract

**Aims:** The overall aim of this project was to encourage mental health patients to participate in the general election. To raise awareness among staff about patient voting rights so they are better equipped to encourage patients to participate.

**Methods:** We conducted face-to-face interviews with all patients throughout the voting process from June to July 2024 on one of the wards.

– Created a page on the trust intranet with information about voting for staff.

– Created a leaflet that was available on the intranet for staff to print out and display.

– Made an educational video on the trust YouTube account and embedded it on the trust intranet.

– Conducted a staff survey after the election to assess knowledge and attitudes about mental health patient voting rights and to evaluate the effectiveness of our interventions.

**Results:** A survey was conducted and was open for 1 month until 18 October 2024. This was circulated to all staff via email distribution lists. We had 18 completed surveys.

– 44% of respondents were vaguely aware or not aware at all of patient voting rights.

– 39% were not confident about which mental health patients could vote in the general election.

– 67% of respondents did not see any of the voting resources.

– 83% were not confident in supporting patients at the general election.

– 88% of staff agreed that the trust should support patients to vote.

– 93% of staff thought it was important for the trust to promote voting rights of patients; however, 5% thought it was not important at all.

– Of all the patients on the ward (N=19), 7 (36.8%) were not interested in voting, 1 was not eligible and 57.9% were already registered.

– Of those registered, 5 (45.5%) made their own arrangements to vote and 2 were supported by the ward to vote (in person and by post).

– 36.4% (4) of patients who had registered to vote and had expressed interest in voting were not able to vote.

**Conclusion:** There is a need to increase awareness of voting rights among both staff and patients. Survey uptake was low, but from the data collected, most staff did not see any of our resources and most are not having conversations with patients about voting rights and the support available to exercise these rights

